# A review of the mechanism, diagnosis, and treatment of *Naegleria fowleri* infection

**DOI:** 10.3389/fmicb.2025.1686695

**Published:** 2025-10-22

**Authors:** Ling Dai, Xin-Ru Guo, Xu-Rui Chen, Ming-Hao Ma, Zi-Han Liu, Juan Lai, Jun Lu, Ming Feng, Xi-Xia Liu, Sheng-Hui Yang

**Affiliations:** ^1^School of Medicine, Hunan University of Chinese Medicine, Changsha, Hunan, China; ^2^School of Nursing, Hunan University of Chinese Medicine, Changsha, Hunan, China; ^3^School of Health, Elderly Care and Nursing, Insurance Professional College, Changsha, Hunan, China

**Keywords:** *Naegleria fowleri*, primary amoebic meningoencephalitis, pathogenesis, diagnostics, therapeutics

## Abstract

*Naegleria fowleri* is a rare pathogen responsible for primary amoebic meningoencephalitis (PAM), a fatal central nervous system infection characterized by rapid clinical progression and an extremely high mortality rate. The existing diagnostic methods are insufficiently sensitive, and therapeutic options are minimal, making early recognition and intervention extremely challenging. This review systematically examines the biological characteristics and pathogenic mechanisms of this pathogen, as well as current diagnostic and treatment strategies, with a particular focus on the groundbreaking applications of emerging technologies such as metagenomic next-generation sequencing (mNGS) in the diagnosis of difficult-to-treat infections. The aim is to provide theoretical support and practical guidance for rapid identification, accurate diagnosis, and timely intervention in clinical practice, serving as a reference for the prevention and treatment of *N. fowleri* infections.

## 1 Introduction

Primary amoebic meningoencephalitis (PAM) caused by *Naegleria fowleri* is characterized by acute onset, rapid progression, severe clinical manifestations, and a mortality rate as high as 97% (Güémez and García, 2021), posing a serious threat to human health. In the early stages of infection, *N. fowleri* primarily causes upper respiratory tract infections, with patients experiencing changes in taste and smell. Subsequently, high fever, nausea, vomiting, and headache, which are signs of meningeal irritation, rapidly develop. Within the next 1 to 2 days, cerebral edema may be induced. Patients often fall into a coma and respiratory failure due to the rapid progression of cerebral edema and increased intracranial pressure, leading to brain herniation. Gharpure et al. (2021) analyzed the case data of 256 patients and found that the median time from symptom onset to death in patients who died from *N. fowleri* infection was 5 days. Moreover, the early symptoms of *N. fowleri* infection are extremely similar to those of bacterial meningoencephalitis, which can easily lead to misdiagnosis in clinical practice (Matanock et al., 2018). Given the rapid progression, high mortality rate, and high likelihood of misdiagnosis of PAM, it is particularly important to develop a standardized and rapid diagnostic protocol. Currently, laboratory diagnosis of *N. fowleri* mainly relies on direct microscopic examination of amoebae in cerebrospinal fluid (CSF), culture, polymerase chain reaction (PCR), and next-generation sequencing (NGS). Direct microscopic examination of CSF is prone to false negatives. PCR can rapidly detect PAM and other types of meningitis, but its appropriate application requires early suspicion of the disease by clinicians. NGS can quickly identify all known infectious diseases with sequenced pathogens, but it is costly. CSF culture is slow and cost-effective, and can be used for postmortem examination to determine the cause of death, but it is not suitable for antemortem diagnosis in infected individuals and may delay treatment; therefore, it is not recommended (Capewell et al., 2015).

Currently, the clinical treatment of *N. fowleri* infection lacks specific and effective drugs. Commonly used drugs in clinical practice, such as amphotericin B, miltefosine, fluconazole, and azithromycin, have limited efficacy and are associated with organ toxicity. The concentration of these drugs reaching the brain parenchyma is limited. For recovering patients, higher doses of drugs are required, which can lead to more pronounced side effects (Chang et al., 2024). Therefore, the development of a clinically efficient therapeutic drug and an efficient drug delivery carrier is of utmost importance. In this regard, the development of new targeted drugs, copper metabolism therapy (Grechnikova et al., 2020), and the development of nanomedicines (Siddiqui et al., 2023; Siddiqui et al., 2020) are popular *N. fowleri* research topics. This article reviews the biological characteristics, infection mechanisms, pathogenic mechanisms, diagnostic methods, and current treatment status of *N. fowleri*, with the aim of providing references and insights for the prevention and control of *N. fowleri*.

## 2 Introduction to *N. fowleri*

### 2.1 The life cycle of *N. fowleri*

*Naegleria fowleri* is a eukaryotic, free-living, thermophilic microorganism that can live freely in water, soil, or hosts, persisting in warm and humid environments. It typically resides in micro-freshwater habitats and the silt of natural water bodies, feeding primarily on bacteria (Siddiqui et al., 2020). Under different natural environmental conditions, *N. fowleri* can exhibit three distinct forms: cyst, flagellate trophozoite, and amoeboid trophozoite (Siddiqui et al., 2016). Current scientific research has confirmed that only the amoeboid trophozoite stage of *N. fowleri* can infect humans and cause PAM. There is no research to date that has proven the other two stages can infect and cause disease in humans. When environmental conditions become adverse, it transforms into a metabolically inactive cyst (Evdokiou et al., 2022). The cyst is highly resistant and can remain dormant at temperatures as low as 4 °C, only commencing reproduction when the temperature increases in the summer (Maciver et al., 2020). However, whether the cysts of *N. fowleri* can cause human disease remains a matter of debate in the academic community. Maciver et al. (2020) conducted a statistical analysis of 336 cases of *N. fowleri* infection, and the results showed that 93% of PAM cases (314 cases) were definitively associated with water exposure. However, there were still 22 cases in which the patients were not described as being infected through water contact. Based on this, they speculated that some of these 22 cases might have been caused by the cysts of *N. fowleri*. Inconsistently, Dorsch et al. (1983) believes that the cases considered to be caused by cysts may have been misjudged due to the failure to fully take into account the presence of *N. fowleri* in domestic water during the research process. Moreover, Evdokiou et al. (2022) conducted animal experiments. They selected mice aged three to 8 weeks and inoculated the cysts of *N. fowleri* into their nasal cavities. After culturing for more than 10 days, the mice were euthanized, and their brain tissues were removed for culture; the infection with *N. fowleri* was negative (Evdokiou et al., 2022). However, the cyst inoculation period in the experiment was relatively short. Some researchers believe that if given enough time to attach to the nasal epithelium, the cysts might be able to complete the infection process (Evdokiou et al., 2022). Whether cysts can infect humans is still unclear. If the cysts of *N. fowleri* can infect humans, the concentration of airborne *N. fowleri* cysts that would be required to cause human infection is unknown. Therefore, longer-term animal experiments involving nasal exposure to air containing cysts are required to provide more definitive and convincing evidence for this conclusion.

### 2.2 The impact of the environment on the survival and distribution of *N. fowleri*

*Naegleria fowleri* is a thermophilic microorganism that can survive within a temperature range of 10–46 °C, with an optimal growth temperature of 40 °C (Aurongzeb et al., 2025; Coronado-Velázquez et al., 2018). Berger (2022) found that the concentration of *N. fowleri* is significantly higher in summer than in winter. With global warming and the increase in human water-based activities, the chances of people coming into contact with *N. fowleri* have increased (Maciver et al., 2020). Additionally, climate change has also led to an increase in *Cyanobacteria* and bacteria in water bodies, which are food for *N. fowleri*, thereby promoting its growth and reproduction (Heilmann et al., 2024). Given the strong survival capacity of *N. fowleri* in high-temperature environments, against the backdrop of global warming, its range of spread is gradually expanding, which may lead to a northward shift in its distribution areas (Russell et al., 2023). As temperatures increase, regions that were previously relatively cold may gradually reach the suitable survival temperature for *N. fowleri*, increasing the likelihood of its occurrence in these areas. These studies indicate that *N. fowleri* exhibits a broad range of temperature adaptability, enabling it to survive in diverse climatic conditions, a characteristic that also makes its spread around the world relatively easy.

*Naegleria fowleri* grows rapidly in freshwater but has not been found in high-salinity marine environments (Stahl and Olson, 2023; Arberas-Jiménez et al., 2024). This indicates that low salinity is conducive to its growth. *N. fowleri* can remain viable for at least 96 h at pH values of 4 to 11, whereas it loses activity within 72 h at pH 3 and within 24 h at pH 12 (Berger, 2022). A pH of 6.5 is the optimal pH for the growth of *N. fowleri* (Stahl and Olson, 2023). Therefore, high concentrations of salt in water, as well as a water pH below 3 or above 11, can all inhibit the survival of *N. fowleri* and can be used as a means of water disinfection.

### 2.3 Epidemiological characteristics

**Figure 1:**
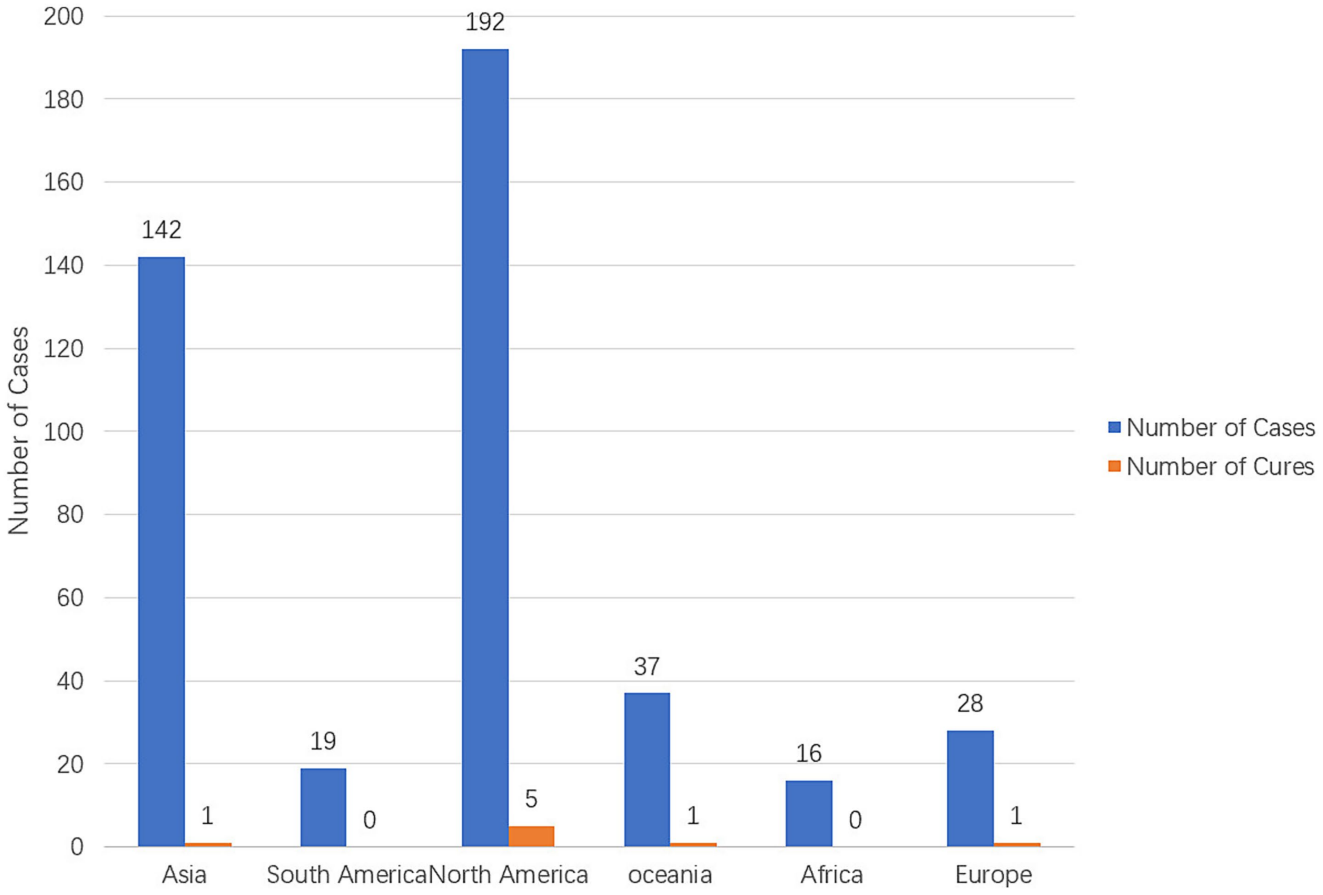
The number of *N. fowleri* cases and cure status in various continents from 1937 to 2024 (Gharpure et al., 2021; Soontrapa et al., 2022; Nadeem et al., 2023; Hall et al., 2024; Cope et al., 2016).

The identified global cases are all concentrated in freshwater recreational areas with higher water temperatures (such as the southern United States of America and Pakistan), with over 80% of infections occurring in the summer (Maciver et al., 2020; Sutrave and Richter, 2021). Climate warming may lead to the expansion of the survival space of *N. fowleri* to temperate regions (Maciver et al., 2020). Among the 11 cases reported in China, 10 were related to outdoor swimming in the hot summer season, indicating that outdoor swimming in summer needs to be a key focus for prevention and control (Chen et al., 2023). Cases of *N. fowleri* infection were systematically retrieved in various continents from 1937 to 2024 in PubMed and CNKI, and the infection and fatal cases in each continent were summarized (Figure 1).

### 2.4 Genomic characteristics and extracellular genetic elements

*Naegleria fowleri* not only possesses a typical nuclear genome, but its mitochondrial genome (mtDNA) and extracellular circular ribosomal DNA (CERE-rDNA) also have unique structures, which may be closely related to its strong environmental adaptability and pathogenic potential. However, despite the accumulation of hundreds of reported infection cases worldwide (Figure 1), as of 2025, the number of complete or high-quality draft genome sequences available in public databases remains extremely limited, with only eight strains, of which only four are considered pathogenic (Aurongzeb et al., 2022). Studies on the clinical isolate AY27 from Pakistan have revealed that its mitochondrial genome is 49,541 bp in length and contains 69 genes (46 protein-coding genes, 21 tRNAs, and 2 rRNAs). Pan-genome analysis indicates that it is of the “open” type (Bpan = 0.137), suggesting that the species has the potential to continuously acquire new genes and adapt to new environments. This capability may be associated with its global dissemination and the maintenance of its pathogenicity (Aurongzeb et al., 2022). In addition, this strain also carries a circular CERE-rDNA with a full length of approximately 15.8 kb. In addition to encoding 18S-5.8S-28S rRNA, it also contains multiple repeat sequences and open reading frames (ORFs). Although its primary function is to encode rRNA, some ORFs can encode hypothetical proteins. Through structural modeling and virtual screening, researchers have identified small-molecule compounds (such as ZINC77564275 and ZINC15022129) that can target and bind to these proteins, suggesting their potential as drug targets (Aurongzeb et al., 2024b).

In the future, with the reduction of sequencing costs and the strengthening of global cooperation, the establishment of an *N. fowleri* genome database that includes strains from different regions and genotypes will provide a crucial molecular basis for the development of rapid diagnostic methods, the exploration of new therapeutic targets, and the understanding of its global transmission dynamics.

## 3 Mechanism of infection

### 3.1 Infection routes of infection

*Naegleria fowleri* is a widely distributed unicellular protozoan that commonly inhabits natural water bodies, such as hot springs, ponds, and freshwater lakes. It is also widely found in untreated tap water and water-based recreational venues, such as swimming pools, and has even been detected in environments, such as hospitals and irrigation channels (Bonilla-Lemus et al., 2020). In daily life, most cases of *N. fowleri* infection in adults and children occur after participation in water-based recreational activities. The trophozoites enter the body through the nasal cavity, causing PAM (Coronado-Velázquez et al., 2018; Flores-Suárez et al., 2024; Jarolim et al., 2000; Cabanes et al., 2001).

### 3.2 The mechanism of *N. fowleri* entering the central nervous system (CNS)

**Figure 2:**
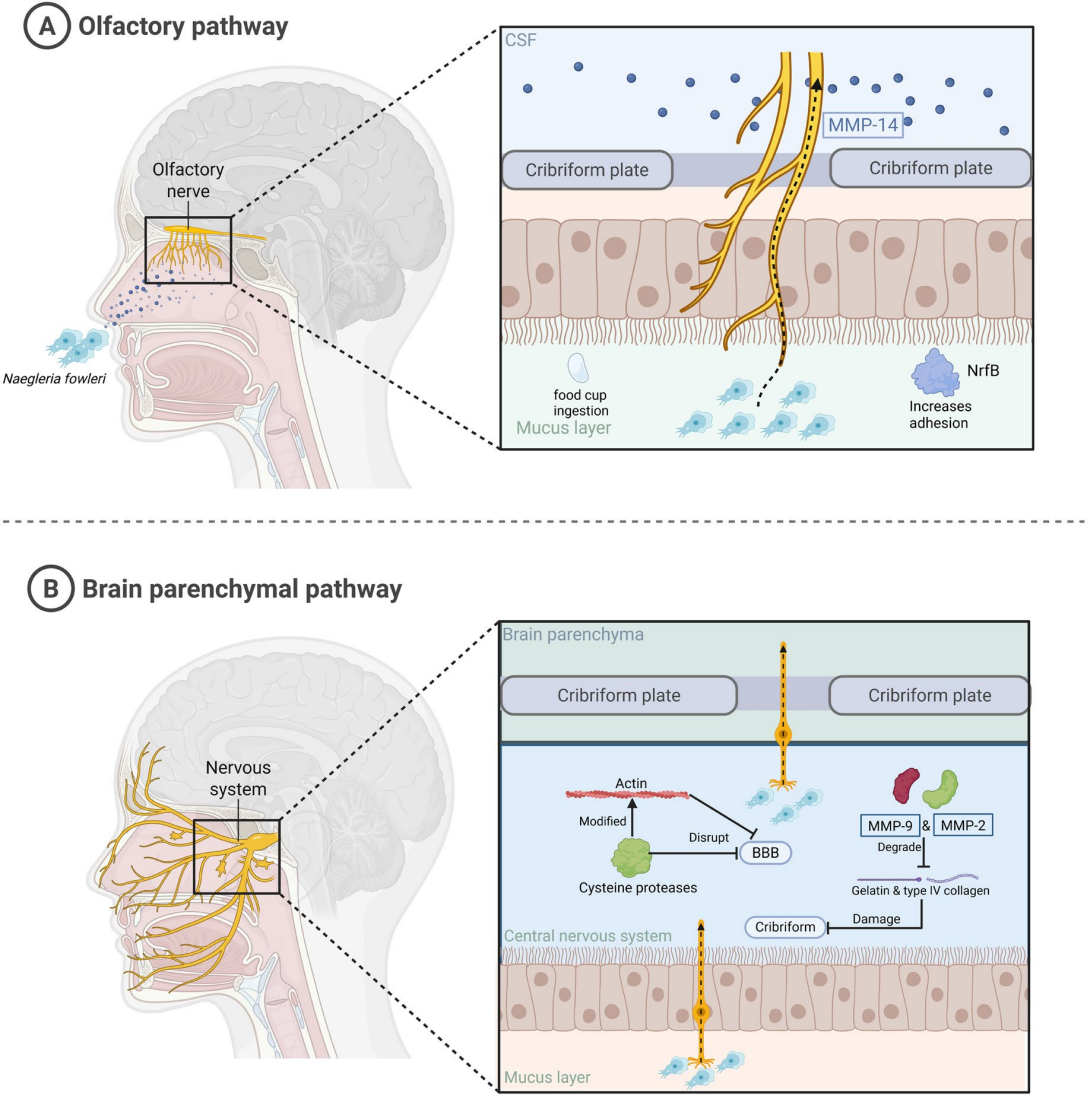
The mechanism by which *N. fowleri* infects humans and enters the brain parenchyma. This figure summarizes the process of *N. fowleri* entering the brain parenchyma. **(A)** *N. fowleri* enters the central nervous system along the olfactory nerve through the nasal cavity. **(B)** *N. fowleri* invades the brain parenchyma.

Many studies have confirmed that humans contract *N. fowleri* through contact with contaminated water, leading to PAM and fatality in patients (Nadeem et al., 2023). Despite the body’s multiple defense mechanisms, the mechanism of *N. fowleri* entry into the nervous system is unclear (Figure 2). Trophozoites of *N. fowleri* adhere to the nasal mucosa, which is the first step in their invasion of the human body. Compared with non-pathogenic amoebae, *N. fowleri* has a higher level of adhesion. In addition, there is a structure on the surface of its trophozoites called the feeding cup, which enables *N. fowleri* to ingest bacteria, fungi, and human tissue (Kim et al., 2024; Jahangeer et al., 2020). Cathepsin B, which is distributed in the feeding cup and pseudopodia, plays an important role in the adhesion process. It is a virulence factor with high immunogenicity (Lê et al., 2022). When it is inhibited, the survival rate increases. The next stage is the invasion into the CNS. The *N. fowleri* trophozoites adhering to the nasal mucosa travel along the olfactory nerve through the cribriform plate to reach the olfactory bulb of the CNS. This stage involves multiple virulence factors. *N. fowleri* trophozoites have at least three types of matrix metalloproteinases (MMPs), among which MMP-2 and MMP-9 are used to degrade gelatin and type IV collagen. However, these two enzymes exist in proenzyme form and need to be activated by MMP-14 to function. The MMPs secreted by *N. fowleri* can degrade the extracellular matrix and destroy the structure of the nasal mucosa and cribriform plate (Que and Reed, 2000). In addition, the cysteine proteases secreted by *N. fowleri* can also cause dislocation and degradation of tight junction proteins between the brain capillary walls of the blood–brain barrier (BBB) and glial cells. This protease can also modify the actin cytoskeleton, thereby altering the stability of the BBB and allowing the amoeba to successfully enter the CNS (Coronado-Velázquez et al., 2018; Lam et al., 2017). Finally, there is the stage where *N. fowleri* enters the brain parenchyma. The trophozoites of *N. fowleri* penetrate the cribriform plate, infiltrate the subarachnoid space, and ultimately reach the brain parenchyma, triggering PAM (Marciano-Cabral and Cabral, 2007).

After summarizing the case data from 1965 to 2018, the majority of infected individuals were male, with a median age of 14 years (Gharpure et al., 2021; Hall et al., 2024). This indicates that patients infected with *N. fowleri* are generally young. This may be related to the higher permeability of the cribriform plate in children or adolescents, which makes it easier for *N. fowleri* to invade the CNS, or it may be related to the fact that the immune system of adolescents has not yet fully developed. However, as for the more specific reasons and mechanisms, further experiments and data provided by researchers are needed to confirm them.

### 3.3 Mechanism of immune evasion

#### 3.3.1 The immune evasion mechanisms of *N. fowleri*

**Figure 3:**
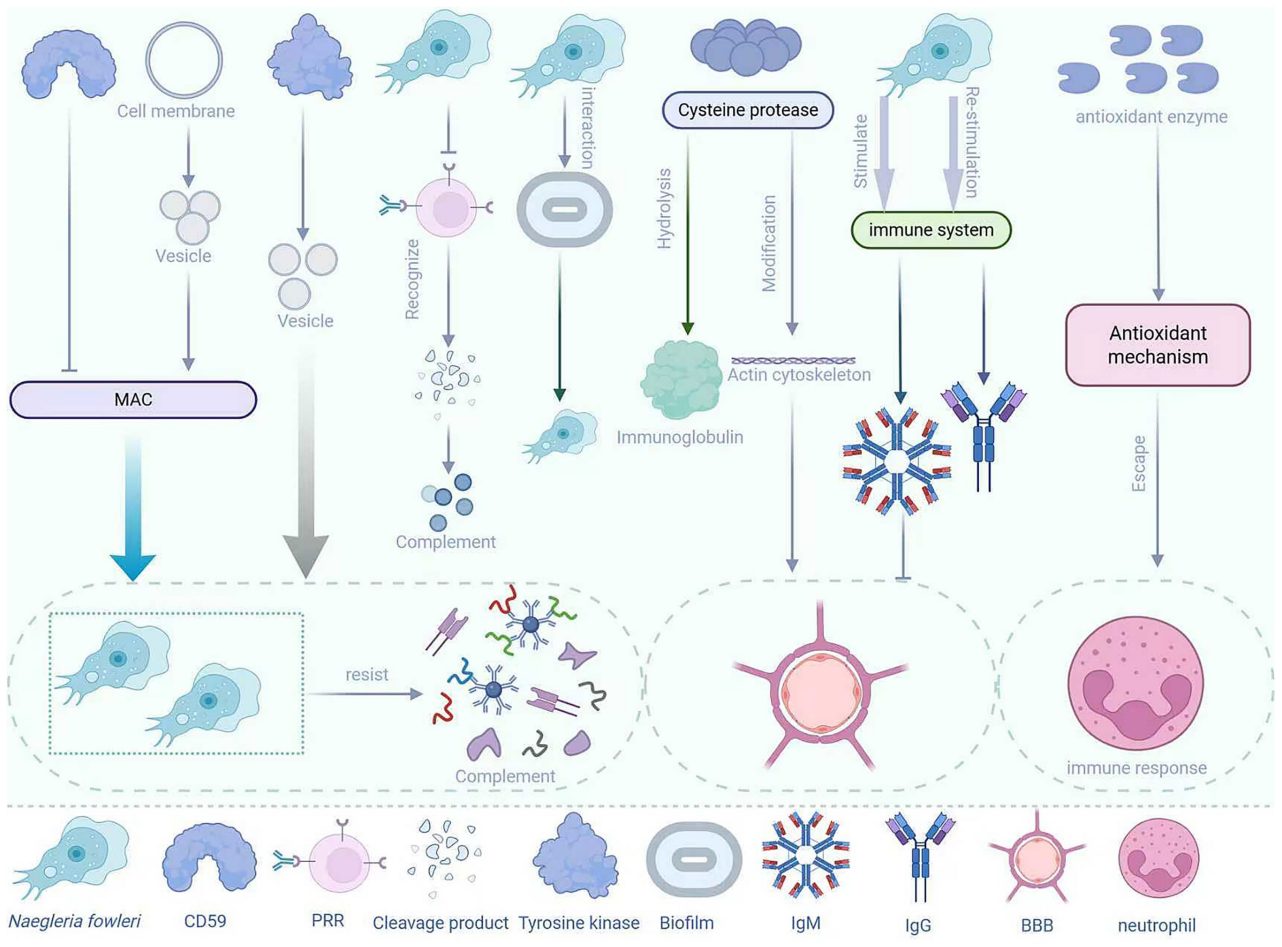
Immune evasion mechanisms of *N. fowleri*.

*Naegleria fowleri* is a pathogen with multiple immune evasion mechanisms, which enable it to survive in the host and cause disease (Figure 3). Under normal circumstances, when the body is first infected with parasites or bacteria and other pathogens, the immune system will rapidly produce IgM antibodies, while IgG antibodies generally only appear during reinfection with the same microorganism (Anam et al., 2024). Therefore, when many people are infected with *N. fowleri*, the first antibodies to appear are IgM antibodies. However, IgM antibodies have a large molecular weight (approximately 900 kDa) and have difficulty crossing the BBB. This natural barrier limits the immune response of IgM antibodies to the pathogen, allowing *N. fowleri* to survive and reproduce relatively “safely” in the CNS (Adelman et al., 1980). When the body fails to prevent the parasite from invading through the olfactory route, the innate response within the epithelium is triggered. However, *N. fowleri* can evade immune detection, penetrate into the lamina propria, and then enter the olfactory nerve bundles (Malych et al., 2025). In addition, Piñero et al. (2019) conducted *in vitro* studies and found that antioxidant enzymes are overexpressed within the trophozoites of *N. fowleri*, indicating that its antioxidant mechanisms can be activated under oxidative conditions to help evade immune responses mediated by neutrophils. The pattern recognition receptors of most mammalian immune cells recognize the degradation products of pathogens, which are usually considered chemotactic factors for immune cell recruitment. However, the trophozoites of *N. fowleri* are resistant to downstream complement-mediated lysis, which is also part of the reason for its immune evasion (Rîpă et al., 2025). Moreover, although the host’s mucin can prevent the trophozoites of *N. fowleri* from adhering to the host’s mucosa, *N. fowleri* often releases proteolytic substances to degrade the host’s mucin, thereby escaping capture by mucin (Rîpă et al., 2025). The lethality of *N. fowleri* infection lies in its ability to rapidly invade the CNS. During this process, the sentinel cells in the respiratory epithelium fail to effectively prevent its invasion and initiate a sufficient immune response (Coronado-Velázquez et al., 2018).

The surface of *N. fowleri* can express a protein similar to human CD59, which can prevent the insertion and activation of the membrane attack complex (MAC), thereby protecting the amoeba from complement-mediated lysis (Fritzinger and Marciano-Cabral, 2004; Cooke et al., 2025). In addition, *N. fowleri* can also shed the MAC (C5b-C9) from the cell surface through the process of vesiculation, thereby protecting itself from complement-mediated lysis (Chu et al., 2002). Moreover, upon contact with complement, *N. fowleri* can activate signaling pathways, such as tyrosine kinase, which are involved in the regulation of vesicle formation and shedding, thereby further enhancing its resistance to complement attacks (Chu et al., 2000). *N. fowleri* can also secrete a series of cathepsin B family enzymes, among which cysteine proteases can modify the actin cytoskeleton of brain endothelial cells (Coronado-Velázquez et al., 2018). This process alters the stability of the BBB, allowing *N. fowleri* to enter the CNS. Among them, the 37 KDa cysteine protease with mucinolytic activity is believed to be associated with the degradation of mucin and evasion of the host immune system (Jahangeer et al., 2020). In addition, cysteine proteases can partially hydrolyze IgA, IgG, and IgM, which also indicates that these enzymes have potential roles in host immune evasion (Rodríguez-Mera et al., 2022; Lee et al., 2014).

#### 3.3.2 The protective role of the biofilm

Biofilms are complex microbial communities formed by bacteria, fungi, and other microorganisms, which adhere to various surfaces through the secretion of extracellular polymeric substances (EPS) (Flemming et al., 2025). *N. fowleri* itself does not form biofilms, but it can interact with biofilms in the environment. First, the bacteria in the biofilm can provide a rich food source for *N. fowleri*, promoting its growth and reproduction (Flemming et al., 2025). Second, EPS, composed of polysaccharides, proteins, extracellular DNA, and lipids, can promote interactions between biofilms and other cells, as well as the adsorption of organic matter, metals, and chemical pollutants. They also promote cell adhesion at the interface and ensure matrix cohesion (Flemming et al., 2025). The biofilm can provide certain protection for *N. fowleri*, enhancing its stress resistance and also facilitating its adhesion during the host invasion process. Moreover, Semenzato et al. (2025) pointed out that some bacteria in the biofilm may interact with amoebae, enhancing their pathogenicity. For example, certain bacteria can survive within amoebae after being phagocytized, which may make the amoebae more invasive (Flemming and Wuertz, 2019; Semenzato et al., 2025). Therefore, biofilms are conducive to the survival and dissemination of *N. fowleri*, which significantly increases the likelihood of its infection in humans.

## 4 Pathogenic mechanisms

### 4.1 Virulence factors of *N. fowleri*

**Figure 4:**
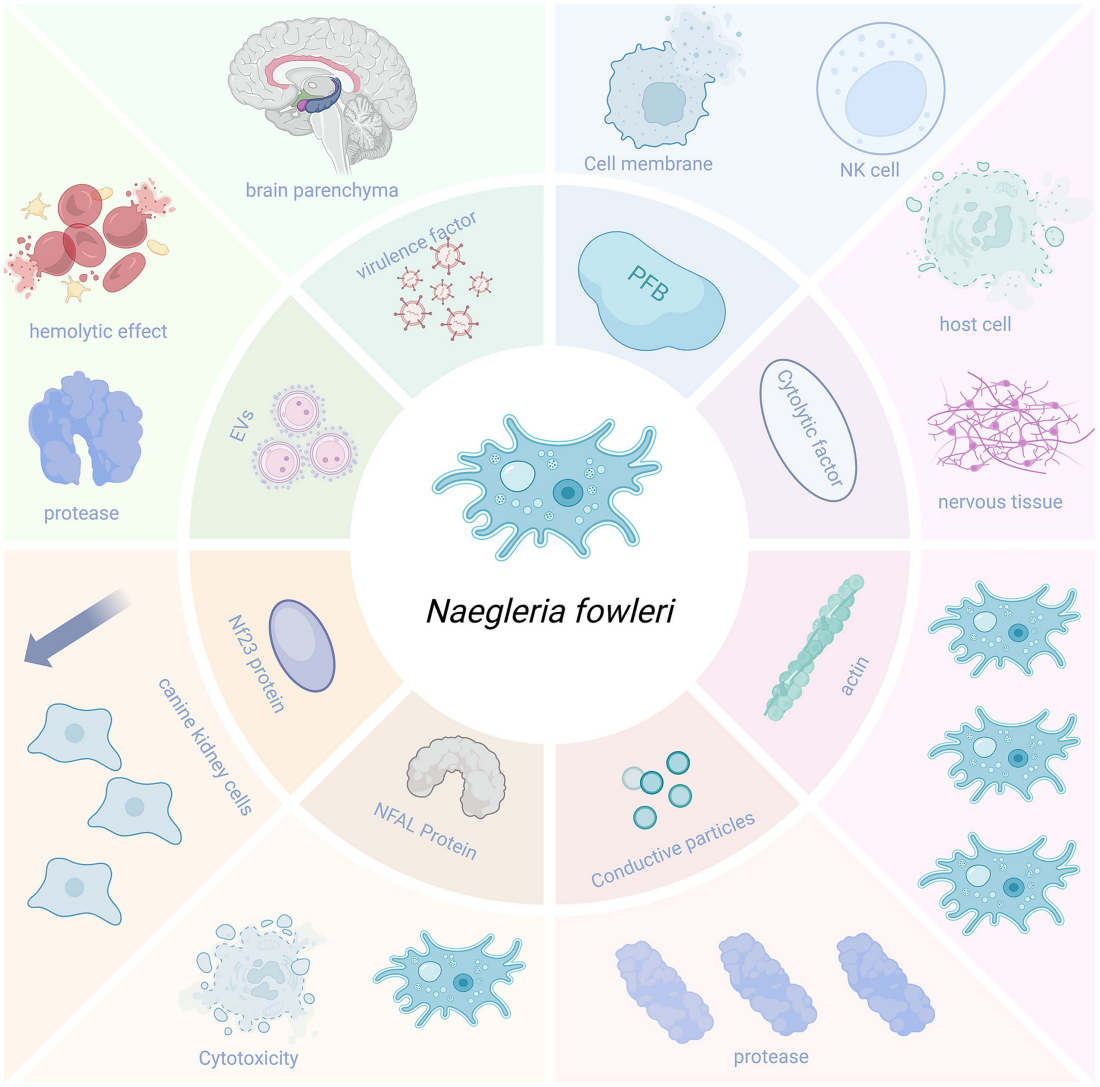
Virulence factors.

*Naegleria fowleri* can secrete a variety of virulence factors that enhance its pathogenicity, facilitating its entry into the brain parenchyma and causing damage to it (Figure 4).

#### 4.1.1 Pore-forming proteins

Pore-forming proteins are membrane complex proteins that can lyse nucleated cells and affect membrane integrity by depolarizing the host cell membrane. *N. fowleri* contains two pore-forming peptides, which have structural similarities to human cytotoxic natural killer (NK) cells and possess strong pore-forming activity, capable of killing both prokaryotic and eukaryotic cells (Young and Lowrey, 1989).

#### 4.1.2 Nfa1 protein

The Nfa1 protein (13.1 kDa) encoded by the *Nfa1* gene found in the pseudopodia of *N. fowleri* is closely related to the amoeba’s formation, adhesion, phagocytosis, and cytotoxicity (Cho et al., 2003). The use of anti-Nfa1 antibodies can reduce the cytotoxicity of *N. fowleri* (Jeong et al., 2004; Shin et al., 2001).

#### 4.1.3 Cytolytic molecules

The pathogenicity of *N. fowleri* also relies on the release of cytolytic molecules, including acid hydrolases, phospholipases, neuraminidases, and phosphatases. These enzymes can destroy host cells and neural tissues, causing brain tissue damage. Moreover, they may be associated with the demyelination observed in the white matter of patients with PAM (Coronado-Velázquez et al., 2018).

#### 4.1.4 Conductance granules

*Naegleria fowleri* contains conductance granules (a cytoplasmic component) with proteolytic activity and lytic function, which may be related to the amoeba’s high levels of collagenase and gelatinase (Coronado-Velázquez et al., 2018).

#### 4.1.5 NF-profilin

NF-profilin is a small actin-binding protein that regulates Nf-actin polymerization and cell motility. NF-profilin is highly expressed in the cell membrane, while Nf-actin is more abundant in pseudopodia, cytoplasm, and food cup structures. This suggests that NF-profilin does not directly participate in the adhesion and phagocytosis of *N. fowleri* but indirectly regulates Nf-actin (Sohn et al., 2025).

#### 4.1.6 Nf23 protein

Flores-Huerta et al. (2020) detected a 23-kDa protein (Nf23) in *N. fowleri*. The expression of Nf23 in *N. fowleri* is higher than that in non-pathogenic microorganisms. Compared with the non-pathogenic species *N. lovaniensis* and *N. gruberi*, the mRNA levels of Nf23 in *N. fowleri* are overexpressed by 4 and 40,000 times, respectively. Flores-Huerta et al. also set up a control experiment, adding anti-Nf23 antibodies and propidium iodide (PI) serum to Madin–Darby canine kidney cells co-incubated with *N. fowleri*. They found that the cytopathic effect was reduced in the group with anti-Nf23 antibodies, indicating that Nf23 may be associated with the virulence of *N. fowleri* (Flores-Huerta et al., 2020). This suggests that Nf23 may be a potential virulence factor, and its antibodies can partially neutralize the amoeba’s cytotoxicity *in vitro*. However, whether it can be developed into a vaccine or therapeutic agent for *N. fowleri* still needs further validation through *in vivo* immunoprotection and challenge experiments.

#### 4.1.7 Extracellular vesicles (EV)

The EVs of *N. fowleri* play an important role in contact-independent pathogenic mechanisms. They exhibit hemolytic activity in red blood cells and can also cause proteolysis, with the proteolytic activity mainly attributed to serine proteases. Additionally, the EVs of *N. fowleri* can enhance paracellular ion permeability and cause damage to MDCK cells (Castelan-Ramirez et al., 2025).

### 4.2 Brain tissue damage induced by the host’s immune response

**Figure 5:**
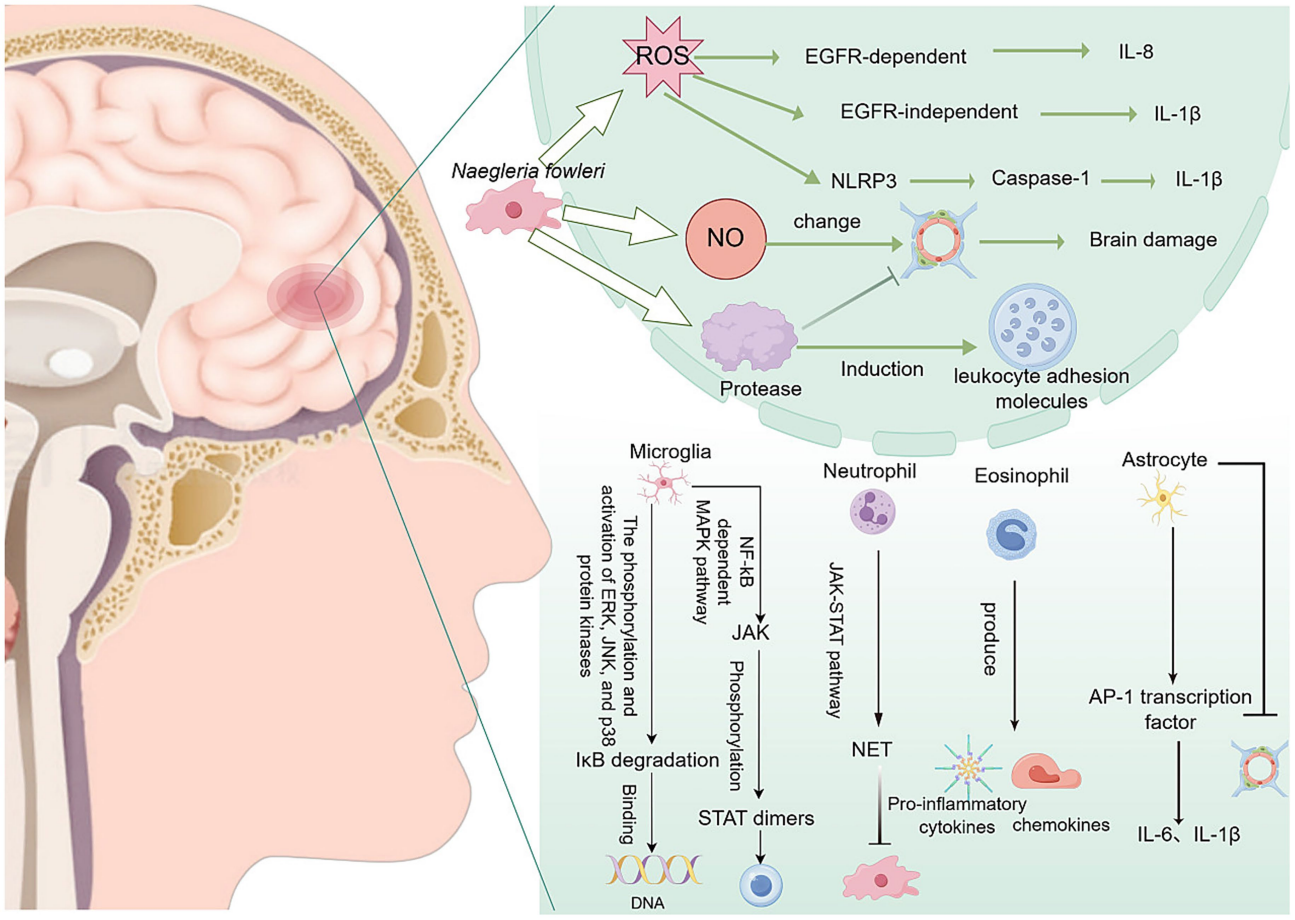
Inflammation and brain damage caused by *N. fowleri* after entering the central nervous system. NET, neutrophil extracellular trap.

After *N. fowleri* invades the CNS, it rapidly activates the host’s immune response centered on reactive oxygen species (ROS) burst, NLRP3 inflammasome assembly, and multiple MAPK/NF-κB signaling axes. The excessive recruitment of immune cells, such as microglia, astrocytes, neutrophils, and eosinophils, leads to a cytokine storm, ultimately resulting in the disruption of the BBB, necroptosis, and irreversible brain parenchymal damage (Figure 5).

#### 4.2.1 Induction of ROS-dependent necrosis

The trophozoites of *N. fowleri* can induce the production of ROS. ROS can activate the epidermal growth factor receptor (EGFR), thereby promoting the production of interleukin-8 (IL-8). Additionally, ROS can induce the expression of the pro-inflammatory cytokine interleukin-1β (IL-1β) through an EGFR-independent mechanism (Jeong et al., 2005). In addition, ROS can also stimulate the formation of the NLRP3 inflammasome, which is a multiprotein complex that can activate caspase-1 and secrete active IL-1β (Kim et al., 2016). Furthermore, *N. fowleri* can activate ROS-dependent necrosis, namely necroptosis, in cells. Necroptosis may be a defense mechanism of the body against *N. fowleri*, but cells are also destroyed, causing irreversible damage to the body itself (Herbst et al., 2002).

#### 4.2.2 Immune cells and inflammatory factors mediate brain tissue injury

After *N. fowleri* enters the CNS, it induces a strong inflammatory response in the body, leading to bleeding and osteolytic necrosis (Jeong et al., 2005). *N. fowleri* triggers a strong inflammatory response through multiple pathways and activates various immune cells, thereby releasing many inflammatory mediators, disrupting the BBB, and causing severe brain tissue damage. This is one of the important pathogenic mechanisms of primary amoebic meningoencephalitis.

##### 4.2.2.1 Microglia-mediated brain tissue injury

Microglia are macrophage-like cells that serve as the vanguard of the CNS immune defense. They are primarily located in the brain and other CNS regions, and their main responsibility is to clear pathogens and damaged neuronal cells (Kaur et al., 2025). When microglia are activated by damage, they can perform antigen presentation and pro-inflammatory functions, thereby initiating an immune response (Kaur et al., 2025). The EVs of *N. fowleri* can induce proinflammatory immune responses in BV-2 microglial cells. This response not only activates the microglial cells themselves but also triggers inflammatory cascades in other brain cells, further infecting host cells (Sarink et al., 2022). In this process, the expression of Toll-like receptor (TLR)-2, TLR-4, and MyD88 is significantly increased. TLRs, as pattern recognition receptors, are important determinants of inflammatory responses and specific downstream intracellular signaling cascades (Sarink et al., 2022). Recombinant *N. fowleri* cathepsin B (rNfCB) promotes the production of pro-inflammatory cytokines, such as tumor necrosis factor *α* (TNF-α), IL-1α, IL-1β, and IL-6 through two major signaling pathways: ① NF-κB-dependent MAPK (Lê et al., 2022; Sarink et al., 2022) and ② JAK–STAT (Sarink et al., 2022; Zhang et al., 2013), thereby inducing pro-inflammatory immune responses in BV-2 microglial cells (Lê et al., 2023). These pro-inflammatory responses not only exacerbate the inflammatory damage and tissue injury caused by *N. fowleri* in the brain but may also trigger a broader immune response. *In vitro* experiments have shown that microglia can release high doses of cytokines, ROS, and reactive nitrogen species (RNS), which are highly neurotoxic to the CNS and can lead to severe tissue damage (Xin et al., 2020).

##### 4.2.2.2 Astrocyte-mediated brain tissue injury

Astrocytes play a crucial role in the CNS, not only participating in maintaining homeostasis but also regulating the immune system. The lysate of *N. fowleri* can activate the AP-1 transcription factor, which induces the expression of IL-1β and IL-6 in astrocytes. The release of these pro-inflammatory cytokines not only further disrupts the integrity of the BBB but also induces immune cells from non-neural sites to enter the brain, thereby triggering an excessive inflammatory response (Kim et al., 2013).

After *N. fowleri* infection, immune cells are recruited to the trophozoites in the olfactory bulb. Eosinophils can produce a variety of pro-inflammatory cytokines and chemokines, such as TNF-*α*, IL-6, IL-8, and eosinophil chemotactic factors. However, although these responses are somewhat helpful for immune defense, they cannot effectively eliminate *N. fowleri*. Additionally, they may further increase leukocyte recruitment and enhance the inflammatory response in the later stages of infection (Lê et al., 2023; Chavez-Munguia et al., 2014; Chen and Moseman, 2022).

##### 4.2.2.3 Neutrophil-mediated brain tissue injury

In PAM, the inflammatory response and associated damage of neutrophils are extremely common. The increase in neutrophil numbers is accompanied by the formation of neutrophil extracellular traps (NETs) and secretion of peroxidase (Jahangeer et al., 2020). Notably, the brain damage caused by the inflammation induced by *N. fowleri* may mainly be due to the host’s excessive immune response, as the degree of damage is relatively mild in brain tissue lacking immune cells (Kim et al., 2016). Neutrophils eliminate pathogens through a variety of mechanisms, including degranulation, proteolytic enzymes, antimicrobial peptides, ROS, and RNS. In the presence of IgA and IgG opsonization and TNF-α, neutrophils can destroy *N. fowleri*. The massive infiltration of neutrophils into brain tissue produces many immune substances, such as immunoglobulins, tumor necrosis factors, and interleukins, causing a severe inflammatory response (Rodríguez-Mera et al., 2022). In addition, *N. fowleri* can induce the formation of NETs. NETs are composed of decondensed chromatin and antimicrobial factors, including myeloperoxidase (MPO), an enzyme stored in the azurophilic granules of immature neutrophils. MPO can catalyze the formation of powerful reactive intermediates, such as hypochlorous acid (HOCl), hypobromous acid (HOBr), hypothiocyanous acid (HOSCN), tyrosyl radicals, and RNS, which play an important role in killing pathogens. However, the massive formation of NETs leads to a severe inflammatory response in the body, but this inflammatory response cannot completely eliminate *N. fowleri* (Carrasco-Yepez et al., 2019). Conversely, it leads to more severe brain tissue damage.

#### 4.2.3 Other factors mediating brain tissue injury

*Naegleria fowleri* exhibits high tolerance to the toxicity of nitric oxide (NO), which enables it to resist the host’s inflammatory response (Jahangeer et al., 2020). The release of NO not only alters the permeability of the BBB, allowing more white blood cells to enter the CNS and further exacerbate brain tissue damage, but also the nitrite produced by NO metabolism is neurotoxic, causing additional damage to the host (Farias et al., 2025). In addition, *N. fowleri* may induce the production of NO-like compounds in brain vascular endothelial cells. Through the interaction between cytokine receptors TLRs, especially TLR4, on endothelial cells, it activates endothelial nitric oxide synthase and inducible nitric oxide synthase, thereby further altering the permeability of the BBB (Coronado-Velázquez et al., 2018).

*Naegleria fowleri* can express a variety of enzymes from the cathepsin B family, including cysteine proteases, which can destroy the structural proteins of host cells, thereby causing tissue damage (Lee et al., 2014). Among them, NfCB (cathepsin B of *N. fowleri*) plays a key role in inducing pro-inflammatory immune responses in BV-2 microglial cells. This pro-inflammatory response may exacerbate the harmful immune response and tissue damage in brain lesions caused by *N. fowleri* infection, thereby leading to PAM. Notably, only structurally and functionally intact NfCB can induce an inflammatory response in the body (Lê et al., 2022). In addition, the cysteine proteases released by *N. fowleri* not only enable it to penetrate the BBB and cause damage but also promote the entry of immune cells into the brain, which is closely related to the extensive production of leukocyte adhesion molecules induced by the trophozoites (Lê et al., 2022).

### 4.3 Potential role of extracellular genetic elements in pathogenicity

In addition to the aforementioned classical virulence factors, recent studies have revealed that the extracellular genetic elements of *N. fowleri*, including mtDNA and CERE-rDNA, may play roles in its pathogenicity that have not yet been fully recognized. First, analysis of the mitochondrial genome of the clinical isolate AY27 indicates that it has an “open” pan-genome characteristic (Bpan = 0.137), suggesting that this pathogen has strong genomic plasticity and may enhance its survival and immune evasion capabilities in the host environment by acquiring new genes or regulatory elements (Aurongzeb et al., 2022). This continuous genomic evolutionary capacity may provide the molecular basis for its adaptation to the unique ecological niche of the human central nervous system and the maintenance of high pathogenicity. Second, although the primary function of its CERE-rDNA is to encode rRNA, its non-ribosomal regions contain multiple ORFs that can encode hypothetical proteins. Through structural modeling and virtual screening, researchers have identified small-molecule compounds (such as ZINC77564275 and ZINC15022129) that can target and bind to these proteins, suggesting that these proteins may have important biological functions (Aurongzeb et al., 2024b). Although their specific functions have not yet been experimentally validated, these hypothetical proteins may be involved in key biological processes such as adhesion, invasion, immune modulation, or metabolic adaptation. Future research may reveal their specific roles in the pathogenic mechanism.

Therefore, these extracellular genetic elements may indirectly enhance the pathogenicity of *N. fowleri* by promoting gene recombination, regulating expression, or encoding proteins with unknown functions. This emerging research direction provides a new molecular perspective for understanding its pathogenic mechanisms and opens up new avenues for developing targeted therapeutic strategies. However, all current hypotheses are based on bioinformatics analysis and urgently need to be confirmed through experimental means such as gene knockout and protein function validation.

## 5 The diagnostic methods of *N. fowleri* infection

**Figure 6:**
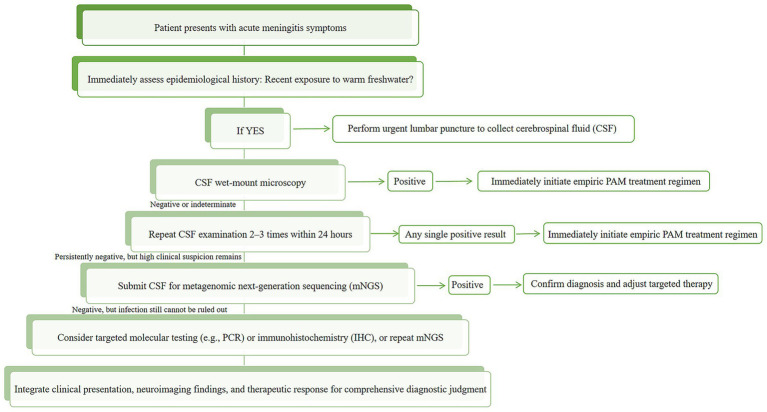
Clinical diagnostic algorithm for suspected *N. fowleri* infection (PAM).

Given the severe clinical challenges posed by *N. fowleri* infections, rapid and accurate pathogen diagnosis is crucial for saving patients’ lives. Therefore, this review systematically elaborates on the current common diagnostic methods for *N. fowleri*, analyzes their respective advantages and limitations (Table 1). The analysis reveals that the probability of diagnosing *N. fowleri* infection using only the current routine clinical diagnostic methods (direct microscopic examination, culture, CT, MRI, etc.) is extremely low. The method with the highest diagnostic yield is mNGS, which can also be used to diagnose other rare infectious diseases, such as Progressive Multifocal Leukoencephalopathy (PML) (Lin et al., 2024). Based on the existing evidence, we have also developed a clinical diagnostic decision pathway for suspected PAM cases (Figure 6). The pathway is designed to guide clinicians to achieve a closed-loop management of “rapid screening-precise confirmation-dynamic evaluation” through a stepwise and progressive diagnostic strategy under the realistic conditions of limited resources and time pressure.

**Table 1:** Diagnostic methods for *N. fowleri* and their advantages and disadvantages.

Diagnostic methods	Types of specimens	Advantages	Limitations	References
Direct microscopic examination	CSF	Preferred, most direct, and fastest	Low detection rate	Guan et al. (2022), Borkens (2024), Zhou et al. (2022), Zaman M. et al. (2025)
Culture	CSF Blood	In hypotonic water, *N. fowleri* rapidly transforms into a flagellate	Time-consuming	Borkens (2024), Schuster (2002), da Rocha-Azevedo et al. (2009)
Computed tomography (CT), Magnetic resonance imaging (MRI)	Intracranial structures and tissues	In late-stage PAM, it can be used to assess brain damage and guide treatment	Non-specific and no abnormalities in early infection	da Rocha-Azevedo et al. (2009), Shaukat et al. (2024)
Immunohistochemical (IHC) staining Indirect immunofluorescence (IIF) staining	Brain tissue	High specificity, high sensitivity, localization, qualitative, and quantitative analysis	There are cross-reactions between species, and there are no specific antibodies	Puthanpurayil et al. (2024)
Enzyme-linked immunosorbent assay (ELISA)	Serum	Convenient and fast	Low sensitivity and specificity, not suitable for timely diagnosis	da Rocha-Azevedo et al. (2009), Martínez-Castillo et al. (2017); Alanazi et al. (2025)
polymerase chain reaction (PCR)	CSF Tissue Blood	Highly specific, sensitive, and rapid	Requires clinical suspicion of PAM	Lin et al. (2024), Puthanpurayil et al. (2024), Baqer et al. (2023), Hong et al. (2023), Che et al. (2023), Maloney et al. (2023), Aurongzeb et al. (2024a), Perez-Perez et al. (2025), Russell et al. (2025), Huang et al. (2024), Phung et al. (2025)
Metagenomic next-generation sequencing (mNGS)	CSF Blood	Rapid, accurate, and capable of diagnosing most rare diseases	Expensive, time-consuming, and susceptible to contamination	Guan et al. (2022), Haston et al. (2020), Gu et al. (2019), Kou et al. (2025), Wei et al. (2024), Song et al. (2024)
Matrix-assisted laser desorption/ionization-time of flight mass spectrometry (MALDI-TOF MS)	Brain tissue	No need for prior culture or subculture, convenient, rapid, and accurate	High technical and sample requirements, and expensive instrumentation	Martínez-Castillo et al. (2017)
Tissue biopsy	Tissue specimens	The gold standard for confirmation	Only applicable for autopsy, not suitable for early diagnosis	Puthanpurayil et al. (2024)

## 6 The current status of treatment for *N. fowleri* infection

Currently, the clinical treatment mainly relies on “old drugs with new uses” such as antifungal or antibiotic drugs like amphotericin B and miltefosine. Although these drugs have achieved therapeutic effects in individual cases, their therapeutic window is extremely narrow. On the one hand, due to the physiological barrier function of the BBB, it is difficult for drugs to reach an effective bactericidal concentration in the brain parenchyma. On the other hand, the high-dose drug administration schemes that are often used to compensate for the insufficient concentration in the brain frequently lead to nephrotoxicity, hepatotoxicity, and neurological side effects, which further exacerbate the patient’s condition (Zhou et al., 2018). To break through this therapeutic impasse, researchers need to advance from two dimensions simultaneously: one is to optimize drug delivery systems, and the other is to innovate drug discovery strategies. The two are complementary and both point to the therapeutic goal of “precision, efficiency, and low toxicity.”

### 6.1 Nanomedicine delivery systems

In terms of drug delivery, nanoparticle-based drug delivery systems have emerged as a highly promising research direction. These systems can actively or passively cross the BBB through various mechanisms to achieve targeted drug enrichment and controlled release in brain tissue, thereby enhancing therapeutic efficacy while significantly reducing systemic toxicity. Currently, the main mechanisms by which nanomedicines penetrate the BBB can be summarized into the following five categories: (i) Adsorptive-Mediated Transcytosis (AMT), where cationic nanoparticles bind to the negatively charged proteoglycans on the surface of BBB endothelial cells through electrostatic interactions, triggering clathrin or caveolin-mediated endocytosis to facilitate transmembrane transport. This mechanism does not require specific ligands and is a versatile delivery strategy due to its operational simplicity (Zheng et al., 2025); (ii) Receptor-Mediated Transcytosis (RMT): The surface of the nanocarrier is modified with a specific ligand that binds to receptors highly expressed on the BBB, triggering receptor-mediated endocytosis and transcellular transport. This is the most mature active targeting strategy currently under research and holds the greatest potential for clinical translation (Liu et al., 2025); (iii) Cell-Penetrating Peptide (CPP)-Mediated Transport: CPPs are a class of short peptides that can transport nanocarriers or drugs across the BBB by directly penetrating the cell membrane or inducing endocytosis. The specific mechanisms are not fully understood and may involve membrane perturbation, endocytosis, or interaction with specific membrane receptors. They have the advantages of high efficiency and low immunogenicity (Nithya and Ramanathan, 2025); (iv) Trojan Horse Approach: Utilizing immune cells such as monocytes and macrophages as “carriers,” nanoparticles are phagocytosed by these cells and then transported to sites of inflammation or infection (e.g., PAM lesions) as the cells migrate, thereby “hitchhiking” past the BBB. This strategy is particularly suitable for brain infections, tumors, or neuroinflammatory diseases and has high lesion targeting specificity (Pavlíčková et al., 2023); (v) Passive targeting in pathological states (Enhanced Permeability and Retention, EPR effect): In pathological conditions such as brain infection, tumor, stroke, or neuroinflammation, the tight junctions of the BBB (e.g., claudin-5, occludin) are disrupted, leading to a temporary increase in permeability. Nanomedicines can take advantage of this “window period” to enter brain tissue through passive diffusion or the enhanced permeability and retention (EPR) effect, achieving local drug accumulation at the lesion site (Aggarwal et al., 2024). All five mechanisms mentioned above can serve as the basis for designing nanocarriers to load anti-amebic drugs (such as amphotericin B, miltefosine, ebselen, etc.), to achieve precise, efficient, and low-toxicity treatment of *N. fowleri* infections. Future research should focus on the synergistic optimization of these mechanisms, the development of intelligent responsive carriers, and preclinical safety evaluations, in order to accelerate their transition from the laboratory to clinical application.

### 6.2 Targeted rational drug design

It is worth noting that the anti-amebic drugs currently used in the clinic (such as amphotericin B and miltefosine) are mostly “old drugs with new uses,” and their discovery processes mostly rely on empirical screening, lacking a deep understanding of the biological characteristics of the pathogen. With the development of genomics, structural biology, and computational chemistry, target-based rational drug design is becoming the mainstream approach in new drug development (Sohail et al., 2025). Comparative Genomics and Subtractive Genomics have become powerful tools for systematically mining novel drug and vaccine targets (Radusky et al., 2015). The core idea of this strategy is to use bioinformatics methods to screen for candidate targets in the pathogen’s “core genome” that simultaneously meet multiple criteria, including “essential for pathogen survival,” “no homology with the host” (to avoid cross-reactions), “located on the cell surface or secreted proteins” (easily recognized by the immune system or targeted by drugs), and “having suitable pocket structures” (capable of binding small-molecule drugs) (Basharat et al., 2022; Dorella et al., 2006). This strategy has been successful in research on other drug-resistant pathogens. For example, in the study of drug-resistant *Salmonella Typhi*, scholars such as Muneeba Afzal analyzed the pan-genome of eight strains and ultimately identified four highly conserved, non-host homologous, and structurally defined enzymes (such as ClpP protease and dihydrofolate synthase folP) as ideal targets. They also identified high-affinity inhibitors from tens of thousands of compounds through virtual screening (Afzal et al., 2023). Similarly, for methicillin-resistant *Staphylococcus aureus* (MRSA), researchers employed a “subtractive” approach, progressively eliminating non-essential and host-homologous proteins from thousands of candidates, ultimately focusing on key targets such as SecY (a protein transport channel). They then screened a library of traditional Chinese medicine compounds, providing new ideas for treating infections caused by drug-resistant bacteria (Khan et al., 2023). Applying this cutting-edge strategy to the study of *N. fowleri* holds great potential. By systematically identifying and validating key targets that are absolutely essential for its survival and can be intervened by drugs (such as unique enzymes in the sterol synthesis pathway, cysteine proteases, mitochondrial function proteins, etc.), we can hopefully develop a new generation of therapeutic drugs with higher efficacy, lower toxicity, and less likelihood of developing drug resistance. In the future, the integration of “target-based rational drug design” with “nanosmart delivery systems”—that is, combining “precise targeting” with “precise delivery”—holds significant promise for overcoming infections caused by *N. fowleri*.

This article systematically reviews the treatment strategies for reported *N. fowleri* infections and research-level drugs currently under investigation. It analyzes the clinically available drugs (Table 2) and preclinical drugs (Table 3) to provide a scientific reference for clinicians to develop individualized treatment plans.

**Table 2:** Drugs already used for clinical treatment.

Drug categories	Drug	Route of administration	Mechanisms of treatment	Limitations	Evidence sources	References
Antifungal drugs	Amphotericin B	Intravenous or intrathecal	Amphotericin B is a class of antifungal drugs that induce rapid leakage of monovalent ions by binding to ergosterol in the cell membrane, eventually leading to cell death. Some studies have shown that amphotericin B induces ultrastructural abnormalities in amoebae, manifested as nuclear deformation, endoplasmic reticulum expansion, reduction of food vacuoles, absence of pseudopodia, mitochondrial abnormalities, increased autophagic vacuoles, and plasma membrane bleeding	Amphotericin B has poor penetration of the blood–brain barrier and requires high doses. Patients treated with Amp B exhibit acute infusion-related reactions and dose-dependent nephrotoxicity, such as nephrotoxicity, anemia, chills, fever, nausea, vomiting, and even brain damage	In vitro data, clinical case reports, and treatment protocols recommended by the Centers for Disease Control and Prevention (CDC)	Grechnikova et al. (2020), Siddiqui et al. (2023), Phung et al. (2025), Fong et al. (2024), Siddiqui et al. (2022), Akbar et al. (2023), Rizo-Liendo et al. (2020), Debnath et al. (2017), Siddiqui and Khan (2023)
	Fluconazole	Intravenous or oral Duration:28 days	Fluconazole is another antifungal agent that acts through a distinct mechanism. As an azole sterol 14α-demethylase (CYP51) inhibitor, it blocks the removal of the C-14 methyl group, thereby depleting ergosterol reserves in the fungal cell membrane. Additionally, fluconazole readily crosses the BBB and rapidly distributes into various regions of the central nervous system	It is associated with adverse side effects; long-term use of azole agents has been linked to hepatotoxicity as well as hormone-related effects, including gynecomastia, alopecia, oligospermia, erectile dysfunction, and hyponatremia	In vitro data, clinical case reports, and treatment protocols recommended by the Centers for Disease Control and Prevention (CDC)	Siddiqui et al. (2023), da Rocha-Azevedo et al. (2009), Hong et al. (2023), Phung et al. (2025), Fong et al. (2024), Siddiqui et al. (2022), Debnath et al. (2017), Rizo-Liendo et al. (2021b), Benitez and Carver (2019)
	Azithromycin	Intravenous or oral Duration:28 days	It interferes with the binding of mRNA to ribosomes, ultimately inhibiting protein synthesis, and exhibits favorable tissue penetration. It has a long half-life, high brain permeability, relatively low toxicity, and potential synergistic effects with amphotericin B (AmB)	It has limited activity against the pathogen and has failed to successfully treat the infection	In vitro study data and animal research case reports	Hong et al. (2023), Phung et al. (2025), Fong et al. (2024), Rizo-Liendo et al. (2021b), Debnath et al. (2018)
Antibiotics	Rifampin	Intravenous or oral Duration:28 days	Easily crosses the BBB	It has limited activity against the pathogen and cannot successfully treat the infection	Clinical treatment cases and combination therapy regimens	Phung et al. (2025), Fong et al. (2024), Siddiqui et al. (2022), Rizo-Liendo et al. (2021b), Debnath et al. (2018)
	Miltefosine	Oral Duration:28 days	Miltefosine is an FDA-approved antileishmanial drug that has demonstrated both in vitro and *in vivo* activity against free-living amoebae (FLA) and has the ability to cross the BBB and accumulate in brain tissue	The most common adverse reactions associated with miltefosine use are severe gastrointestinal side effects, such as vomiting, nausea, abdominal pain, and diarrhea, and there is a risk of developing drug resistance. Additionally, there is a possibility of permanent brain damage or other disabilities	Clinical treatment cases and animal research case reports	Siddiqui et al. (2023), Hong et al. (2023), Phung et al. (2025), Fong et al. (2024), Siddiqui et al. (2022), Debnath et al. (2018), Guerlais et al. (2024), Pijpers et al. (2019)
Corticosteroids	Dexamethasone	Intravenous	Dexamethasone is commonly used to reduce intracranial pressure and control cerebral edema, actively managing brain swelling	It is mainly used as an adjunctive therapy to control cerebral edema and has minimal effect on *N. fowleri*	In vitro study data, clinical treatment cases, and combination therapy regimens	Fong et al. (2024), Siddiqui and Khan (2023), Rizo-Liendo et al. (2021b)

**Table 3:** Drugs in preclinical research.

Category	Drug	Research models
Antifungal drugs (CYP51 inhibitor)	Posaconazole (Debnath et al., 2017) Isavuconazole (Debnath et al., 2017)	In vitro models
SMT inhibitors	Cyclopamine (Abraham et al., 2022) Chelerythrine (Abraham et al., 2022) Berberine (Abraham et al., 2022) Tanshinone 2 A (Abraham et al., 2022) Catharanthine (Abraham et al., 2022)	In vitro models, animal models
ERG2 inhibitors	Tamoxifen (Zhou et al., 2018)	In vitro models, animal models
Farnesyltransferase inhibitors	Lonafarnib (Zhou et al., 2018)	In vitro models, animal models
Statin	Pitavastin (Baig, 2020)	In vitro models
	Fluvastatin (Rizo-Liendo et al., 2020) Atorvastatin (Rizo-Liendo et al., 2020) Cirivastatin (Baig, 2020) Rosuvastatin (Baig, 2020) Rosuvastain (Rizo-Liendo et al., 2021b) Pitavastatin (Rizo-Liendo et al., 2021b) Cetvastatin (Rizo-Liendo et al., 2021b)	In vitro models
polyene antibiotic	Coriffennet (Debnath et al., 2012)	In vitro models, animal models
Indolecarbazide	Sarcocystin	
Synthesis of organic selenium drugs	Ebselen (Debnath et al., 2018)	In vitro models
Phenyl vinyl sulfone-related compounds	BAY11-7082 Debnath et al. (2017) BAY11-7085 Debnath et al. (2017)	In vitro models
antirheumatic	Auranofin (Escrig et al., 2020)	In vitro models, clinical study models
Enolase inhibitors	ENO inhibitors (Milanes et al., 2024) HEX (Milanes et al., 2024)	In vitro models, animal models
Fatty acid oxidation inhibitor	Etomosier (Sarink et al., 2020) Perhexylamine (Sarink et al., 2020)	In vitro models, clinical study models
Antiepileptic drugs	valproic acid (Sarink et al., 2020)	In vitro models, clinical study models
Antipsychotics	Thioridazine (Sarink et al., 2020)	In vitro models, clinical study models
Semi-synthetic macrolide antibiotics	Rocomycin (Kim et al., 2008) Roxithromycin (Kim et al., 2008)	In vitro models, animal models
Copper ion carrier	Ebocillin (Grechnikova et al., 2020)	In vitro models
Naphthyridine derivatives	Saturated 1, 6-naphthyridinequinazolinone(Rizo-Liendo et al., 2021a)	In vitro models, animal models
Isobenzofuran-1 (3H) -one derivatives	Novel isobenzofuranone (Rizo-Liendo et al., 2021b)	In vitro models, animal models
S-Adenosyl-L-homocysteine hydrolase (NfSAHH)	Heliocide H2 (Zaman A. et al., 2025; Shah et al., 2025) Curcumin (Zaman A. et al., 2025; Shah et al., 2025) Piceid (Zaman A. et al., 2025; Shah et al., 2025)	In vitro models
Serine carboxypeptidase ligand	SAR1 (Madero-Ayala et al., 2022)	In vitro models

## 7 How to prevent infection with *N. fowleri*

### 7.1 Water disinfection

Water disinfection is of substantial importance for the elimination of *N. fowleri* infections. Conventional disinfection methods are divided into physical and chemical disinfection. Presently, the most commonly used method in swimming pools and other recreational places is chemical disinfection, which involves using disinfectants at a certain concentration to achieve the disinfection effect. The use of different types of disinfectants has different inhibitory effects on the activity of *N.* fowleri (Hu et al., 2025). Hu et al. (2025) compared the disinfection effects of four disinfection methods using chlorine dioxide (ClO₂), free chlorine, chloramine, and ultraviolet (UV) light combined with chlorine agents in drinking water distribution networks. They concluded that using ClO₂ as a disinfectant has the highest inactivation efficiency, with a *Ct*99% of 1–5 mg·min/L. Using free chlorine as a disinfectant requires a higher concentration to effectively inactivate the trophozoites of *N. fowleri,* and biofilms can enhance its chlorine resistance. Chloramine has an even lower inactivation efficiency, and the highest number of *N. fowleri* gene copies was detected in chloraminated distribution networks among the four disinfection methods (Hu et al., 2025). In addition, Sarink et al. (2020) found that in mixed biofilms of *Escherichia coli* strains cultured under field and laboratory conditions, *N. fowleri* can survive for 7 days with intermittent dosing of chlorine at 0.6 mg/L. However, when associated with biofilms in drinking water systems, the survival rate of *N. fowleri* is more than 30 times the recommended chlorine concentration in drinking water (20 mg/L for 3 h). This indicates that compared with biofilms in field and laboratory environments, *N. fowleri* shows stronger chlorine resistance when forming biofilms in drinking water environments (Sarink et al., 2020). This indicates that different disinfectants have different inactivation rates for *N. fowleri*, and the differences in biofilms formed under different environmental conditions can also lead to different resistance levels of *N. fowleri* to the same disinfectant. In large recreational facilities, physical disinfection methods are rarely used alone. Combining UV light with chlorine agents for disinfection can significantly eliminate *N. fowleri* trophozoites in water, reduce the amount of chlorine agents used, and lower the risk of harm to humans from residual chlorine at high concentrations in the water (Arberas-Jiménez et al., 2022). Additionally, boiling can also eliminate *N. fowleri* in water.

### 7.2 Avoiding risky behaviors

In the warmer summer months, when choosing a swimming area, priority should be given to well-maintained, clean, and regularly disinfected sites, such as swimming pools. Avoid swimming or washing your face in stagnant waters or ponds. After swimming, it is recommended to rinse the nasal mucosa and nasal cavity with distilled water or pure bottled water. Additionally, during swimming, try to avoid stirring up sediments in the water, as the bottom sediment layer of water bodies contains a large amount of decayed matter that may harbor *N. fowleri*. To prevent direct contact of contaminated water with the nasal mucosa, using a nose clip can be a preventive measure (Rizo-Liendo et al., 2021a). When performing certain religious activities that require nasal cleansing, it is recommended to use clean containers and boiled water, or commercially available distilled water or pure water for this activity (Alanazi et al., 2025; Miller et al., 2015).

Given that the diameter of *N. fowleri* trophozoites is approximately 10–25 μm and that of cysts is around 7–12 μm, their physical size is much larger than that of common bacteria and viruses (Evdokiou et al., 2022). Therefore, in addition to chemical disinfection, intercepting *N. fowleri* with physical barriers is an effective supplementary means to control its spread in public places, especially artificial water bodies. This can be achieved through the following methods: (i) Filtering systems: Install micron-level filters in the recirculation systems of recreational facilities; (ii) Sediment management: Regularly clean the sediment at the bottom of swimming pools; (iii) Water flow and recirculation filtration: Ensure that the water in all areas is effectively recirculated and filtered; (iv) Water source treatment: For swimming pools relying on natural sources (rivers, lakes, etc.), enhance physical barriers such as sedimentation and filtration.

## 8 Future perspectives

PAM caused by *N. fowleri* poses a severe challenge to global public health due to its extremely high mortality rate and rapid disease progression. Although some progress has been made in diagnosis and treatment in recent years, the current prevention and control system still faces many bottlenecks. In terms of diagnosis, although emerging technologies represented by mNGS have greatly improved the ability to identify this rare disease and have become an important tool for screening encephalitis of unknown etiology, their high cost and dependence on specialized equipment severely limit their widespread application in primary medical institutions and resource-poor areas. At the same time, traditional microscopic examination and culture methods are not sensitive enough or take too long to meet the urgent clinical need for early and rapid diagnosis. Future research urgently needs to focus on developing more economical, convenient, and highly sensitive point-of-care rapid diagnostic techniques, such as optimizing matrix-assisted laser desorption/ionization-time-of-flight mass spectrometry (MALDI-TOF MS), to move it from laboratory research to clinical practice and thus win valuable golden time for patients’ treatment.

In treatment, current reliance on drugs such as amphotericin B and miltefosine is suboptimal, with significant side effects like anemia, fever, nausea, and nephrotoxicity. Nanomedicine delivery systems, though promising for crossing the blood–brain barrier, are still in preclinical stages. Future drug development must adopt a “dual-track” strategy: accelerating nanocarrier safety evaluation and clinical translation for precise drug delivery, and pushing rational drug design based on targets. Using comparative and subtractive genomics, we aim to find unique, essential drug targets in *N. fowleri* for developing new, effective, low-toxicity, and less-resistant drugs. In pathogenic mechanism research, while we have identified several virulence factors and host immune responses, current *in vitro* models cannot fully simulate *N. fowleri* ‘s behavior *in vivo*, limiting our understanding. For prevention, we should enhance monitoring and intervention in high-risk areas and raise public awareness of *N. fowleri* infection prevention.

In summary, effectively addressing PAM caused by *N. fowleri* hinges on breaking through the bottlenecks in the entire chain of “diagnosis-treatment-prevention.” At present, although mNGS has accelerated diagnostic speed, nanodelivery technology has shown potential for crossing the blood–brain barrier, and physical–chemical combined prevention and control strategies are being advanced, each link still faces the core challenges of “difficult translation, high cost, and unclear mechanisms.” The focus of future research should be on “translational medicine” and “precision prevention and control.” On one hand, it is necessary to transform basic research findings (such as the functional interpretation of CERE-rDNA, mtDNA, etc.), the host-pathogen interaction network, and unique metabolic pathways into practical tools such as high-sensitivity point-of-care diagnostic reagents and smart nanomedicines designed based on targets. On the other hand, there is an urgent need to build a closed-loop research system integrating “laboratory-clinical-field,” and accelerate the process from mechanism exploration to clinical validation and then to policy implementation through interdisciplinary collaboration. Only in this way can we improve the survival rate and quality of life of patients with *N. fowleri* infections and bring more hope and protection to those threatened by this infection.
